# Assisting the individuals with the burden of concealment

**DOI:** 10.1192/j.eurpsy.2026.10369

**Published:** 2026-07-31

**Authors:** K. Başar

**Affiliations:** Department of Psychiatry, Hacettepe University, Ankara, Türkiye

## Abstract

**Abstract:**

Sexual and gender minorities often face stigma and discrimination from the time they start exploring their identities, even if they don’t choose to diclose this to others. They may experience negative attitudes in almost all social contexts, starting from the closest one including the family, if they don’t conform with the expected gender roles. Finallly, even if they work hard not to reveal their identity in their appearance, behavior or attire, in addition to the mental effort load related to this nonstop effort, they have to carry the burden of the expected stigmatization and discrimination. In many contexts, nondiclosure may appear protective mentally; it has been shown to be so especially in societies with higher levels of stigmatization. But in all social contexts, concealment of an identity comes with a burden, especially when its control is not on the individual but it is forced on the individual by the social environment. Mental health professionals should be famiiar with the threats existing in the society for the individual, but also should have the capacity to work on the individual’s use of concealment as a defense strategy. Since when it is used excessively and rigidly, this defense can itself be a source of stress. Furthermore, it may lead to missing the authentic potential of the individuals in their personal, interpersonal, and professional domains. In addition to increasing the capacity to use disclosure and concealment interchangeably and to the benefit of the individual, the professional should work on possible resources of psychological resilience for the individual. These efforts often involve the improved relationships with peers from the same or similar minorirty groups, peers from the general population, and family members.

**Disclosure of Interest:**

None Declared

